# Shear Stress as a Major Driver of Marine Biofilm Communities in the NW Mediterranean Sea

**DOI:** 10.3389/fmicb.2019.01768

**Published:** 2019-07-31

**Authors:** Elisa C. P. Catão, Thomas Pollet, Benjamin Misson, Cédric Garnier, Jean-Francois Ghiglione, Raphaëlle Barry-Martinet, Marine Maintenay, Christine Bressy, Jean-François Briand

**Affiliations:** ^1^Laboratoire MAPIEM (EA 4323), Université de Toulon, Toulon, France; ^2^UMR BIPAR, INRA, ANSES, ENVA, Université Paris-Est, Maisons-Alfort, France; ^3^CNRS/INSU, IRD, MIO UM 110, Mediterranean Institute of Oceanography, University of Toulon – Aix-Marseille University, La Garde, France; ^4^CNRS, Sorbonne Université, UMR 7621, Laboratoire d’Océanographie Microbienne, Banyuls-sur-Mer, France

**Keywords:** marine microbiome, multi-species biofilm, hydrodynamic, artificial surface, Sphingomonadaceae

## Abstract

While marine biofilms depend on environmental conditions and substrate, little is known about the influence of hydrodynamic forces. We tested different immersion modes (dynamic, cyclic and static) in Toulon Bay (north-western Mediterranean Sea; NWMS). The static mode was also compared between Toulon and Banyuls Bays. In addition, different artificial surfaces designed to hamper cell attachment (self-polishing coating: SPC; and fouling-release coating: FRC) were compared to inert plastic. Prokaryotic community composition was affected by immersion mode, surface characteristics and site. Rhodobacteriaceae and Flavobacteriaceae dominated the biofilm community structure, with distinct genera according to surface type or immersion mode. Cell density increased with time, greatly limited by hydrodynamic forces, and supposed to delay biofilm maturation. After 1 year, a significant impact of shear stress on the taxonomic structure of the prokaryotic community developed on each surface type was observed. When surfaces contained no biocides, roughness and wettability shaped prokaryotic community structure, which was not enhanced by shear stress. Conversely, the biocidal effect of SPC surfaces, already major in static immersion mode, was amplified by the 15 knots speed. The biofilm community on SPC was 60% dissimilar to the biofilm on the other surfaces and was distinctly colonized by Sphingomonadaceae ((*Alter)Erythrobacter*). At Banyuls, prokaryotic community structures were more similar between the four surfaces tested than at Toulon, due possibly to a masking effect of environmental constraints, especially hydrodynamic, which was greater than in Toulon. Finally, predicted functions such as cell adhesion confirmed some of the hypotheses drawn regarding biofilm formation over the artificial surfaces tested here.

## Introduction

Marine bacteria colonize any submerged surface in a matter of seconds and form complex biofilms over time (Dang and Lovell, 2016), as defined by the cell attachment and production of a hydrated polymeric matrix that allows aggregation (Costerton et al., 1995). The evolution of biofilm colonization depends on several factors, the most investigated being water chemistry and substrate surface properties. Hydrodynamics appears to be a structuring factor for ocean life and is scarcely studied regarding biofilms. Challenging the attachment of organisms with flow has been tested mainly in the laboratory and/or with freshwater communities, where flow-mediated changes affect community richness (Besemer et al., 2007), with variable biofilm diversity according to time of incubation (Besemer et al., 2007; Rochex et al., 2008) and flow speed (Douterelo et al., 2016). Core community was better related to shear stress than to original microbial groups in stream or floodplain ecosystems (Niederdorfer et al., 2016), and biofilm thickness was affected by velocity (Battin et al., 2003). In marine ecosystems, only diatom communities have been studied, revealing a change in both cell number and composition (Zargiel and Swain, 2014; Nolte et al., 2018). This suggests that hydrodynamic stress influences biological settlement on marine surfaces, but little is known about the impact of shear stress on prokaryotic biofilm development in natural conditions. Under static mode, communities tend to converge over time on Non-active substrates such as plastic, with early domination by γ- and α-Proteobacteria and Bacteroidetes (Lee et al., 2008; Elifantz et al., 2013; Briand et al., 2017), even though microbial communities vary according to surface type and site when incubated *in situ* (Lee et al., 2014; Briand et al., 2017).

After microbial settlement, marine biofilms entail many costs for industrial systems, such as biofouling, but also represent ecological roles for larval settlement, elemental cycling and cell protection from contaminant concentrations (Dang and Lovell, 2016; de Carvalho, 2018). The latter can be particularly important in coastal areas, which are often contaminated by trace metals due to anthropogenic activities. These impose environmental stress, causing specific community structure shifts in ultraphytoplankton (Coclet et al., 2018), benthic (Misson et al., 2016) and planktonic prokaryotic communities (Misson et al., 2016; Coclet et al., 2018, 2019). In addition to environmental drivers, biofilm succession varies with surface physical characteristics (Salta et al., 2013). Stochasticity (or selection) for the early attachment of species to form a biofilm can be affected not only by chemical composition or surface properties (Webster and Negri, 2006; Jones et al., 2007; Lee et al., 2008). Considering chemical composition, biocide-containing surfaces are the most studied for their impact on microbial community density and diversity (Briand et al., 2017). Furthermore, physical properties such as low-energy surfaces tend to allow the formation of weaker chemical interactions (Lejars et al., 2012), which should lead to higher release rate of cells under flow, or lead to an increased abundance of taxa with greater adhesion force, as shown by short-term immersion studies (Michael et al., 2016). However, little is known about the surface effect on the selection of microbial communities after long-term immersion.

Contrasting experimental conditions allowed us to identify the influence of the hydrodynamic mode on biofilm microbial communities on the French Mediterranean Sea coast. The effect of hydrodynamics was tested specifically by comparing one static to two dynamic (continuous or intermittent rotation) immersion modes. Colonization was followed from day one for 1 year. We used three different substrate surfaces: one reference considered as inert (poly (vinyl chloride), PVC) and two types of commercial antifouling coatings. Four questions were addressed in this study: (1) the effect of high shear stress on cell count under dynamic mode; and (2) the variation in biofilm community structure over time; (3) later, long-term immersion was assessed under the hypothesis that microbial communities would differ in biofilm maturation imposed by the different immersion types. We hypothesized that hydrodynamics would play a greater role than variable surface characteristics and tested the combined effect of both immersion and surface types on communities; and (4) finally, static immersion was evaluated in two sites within the Mediterranean Sea to observe the local effect on biofilm community structure. Biofilm microbial communities were quantified by flow cytometry, and prokaryotic communities (16S rRNA sequencing) were studied with discriminating approaches (LeFSE) and network analysis. Predictive functional analyses were also performed in order to expand the community structure approach.

## Materials and Methods

### Experimental Design and Sampling

Biofilm development was studied over three types of surfaces: sand-blasted PVC as a reference surface and three commercial antifouling (AF) coatings, including two fouling release coatings (FRC1 and 2) and one self-polishing coating (SPC).

Sandblast was performed to increase the roughness of the PVC and consequently the attachment process providing a reference to biofilm formation. FRCs are surfaces with a low surface free energy and elastic modulus that prevent the adhesion of organisms and enhance the fouling release property. FRC1 is an ambiguous smooth surface composed of a poly (dimethylsiloxane; PDMS)-based elastomer and an amphiphilic additive, which is able to diffuse at the surface to provide both hydrophilic and hydrophobic properties (Duong et al., 2015) that disturb the settlement of marine organisms; FRC2 is composed of a hybrid epoxy/polysiloxane surface. SPC contains biocides, mainly copper derivatives (copper oxide, zinc oxide, zineb and copper pyrithione) here, which are released into the seawater in constant mode.

Static panels were represented by 5 × 5 cm of PVC, covered or not by the AF coatings, while for dynamic incubation, panels of PVC or anticorrosive-protected steel covered with the AF coatings with dimensions 15 × 3.5 cm and curvature radius of 5.07 cm were used (ETS Lorton; Pessac).

Panel immersion was achieved in dynamic mode by a rotor directly immersed in seawater (43°06”18.8” N; 5°53”7.7”E; Supplementary Figure S1) and in static mode with a raft [43°06”25”N; 5°55”41”E; (Briand et al., 2012)], both in Toulon Bay (eastern French Mediterranean Sea). Additionally, panels were immersed in the vicinity of the rotor for only 5- and 75-days comparison with PVC. In both structures, panel disposition was random for minimal impact of depth, which varied from 0.3 to 2 m (photic zone). Dynamic immersion panels were disposed over a 1 m diameter rotor, with shear force uniform between the panels, and a rotation speed of 15 knots. In addition to the continuous rotation mode (further referred to as dynamic mode), a cyclic mode was established with a succession of 2 months of static mode immersion followed by 15 days in dynamic mode for 1 year, to mimic ship activities (Marceaux et al., 2018).

Colonization under dynamic mode was assessed after 1, 5, 12, 32, and 75 days in Toulon Bay, starting on the 26th May 2016. A long-term assessment was performed to compare dynamic, cyclic, and static modes in Toulon Bay, from February 2016 to February 2017, with a single sampling after 365 days of immersion. Static mode was also performed at ∼500 m offshore of Banyuls-sur-Mer, north-western Mediterranean Sea (NWMS) from May 2015 to May 2016 using a framework attached to the SOLA buoy (42°29”N; 03°08”E). In total, 114 panels were immersed for the dynamic mode assessment: 90 on the rotor (3 surfaces × triplicate × 5-time points × 2 analyses as in flow cytometry and DNA extraction); and 24 PVC panels in the static mode near the rotor (1 surface × triplicate × 4-time points × 2 analyses as in flow cytometry and DNA extraction). For the long-term immersion experiment, 96 panels were immersed (4 surfaces × triplicate × 1 time point × 4 immersion modes × 2 analyses).

Water parameters and surface characteristics (roughness, waviness and wettability) were described and expressed in the Supplementary Tables S1, S2, respectively.

### Sample Treatment for Cell Density and DNA Extraction

A set of three panels was used for flow cytometry and another set for DNA extraction for each condition. Each panel was scraped with a sterile scalpel, and biofilms were fixed in 10 mL solution of artificial seawater (ASW; Sigma-Aldrich) containing 0.25% glutaraldehyde (Sigma-Aldrich). Fixed biofilms for flow cytometry and scraped biofilm for DNA extraction were kept at −80°C until analysis. Raw flow cytometry datasets are available on request. It was not possible to assess the percentage of cell detachment by the scalpel, but low standard errors between panels in biological replicates highlight the reproducibility of the technique, as presented elsewhere (Briand et al., 2017).

Flow cytometry measurements for cell count, and DNA extraction with PowerBiofilm DNA isolation kit (Qiagen) have been described previously (Pollet et al., 2018). Seawater (1 L) was successively filtered at 3 and 0.2 μm before DNA extraction (Pollet et al., 2018). 16S rRNA gene was amplified with the 515F-Y/926R primers set (Parada et al., 2016) by the method described previously (Pollet et al., 2018). When no amplification was obtained from all three replicates, samples were pooled before amplification (all surfaces from dynamic and cyclic immersions). Equimolar mixes of amplicons were sequenced using MiSeq Illumina 2 × 250 pb chemistry and generated over 1.2 million raw reads. After quality control all samples were rarefied to 10,850 reads for further analysis. Sequences assessed in this study have been submitted to NCBI under the accession number PRJNA504753.

### Data Analysis

Raw sequences were cleaned with QIIME and Prinseq-lite (Schmieder and Edwards, 2011) and further analyses were performed for OTU clustering at 97%. Taxonomy assignment and alpha-diversity indexes were calculated with QIIME (version 1.9.1), and Silva database version 132 (Caporaso et al., 2010).

The BIOM table after conversion was exported to R and treated with *vegan*, *Tax4Fun*, *ggplot2*, *reshape* and dependent packages for graphical representation and statistical analysis. Total bacteria count, α-diversity and predicted functions were compared between surfaces and/or time or site with one or two-way ANOVA with R (version 3.4.2). Due to the absence of replicates for dynamic incubation, comparison was performed between surfaces assuming different times as replicates.

Chao1, Simpson and Faith’s phylogenetic diversity (PD) (Faith and Baker, 2007) indices were calculated in QIIME to display α-richness and α-diversity of samples, respectively. Pielou evenness index was calculated as the Simpson index/ln (observed OTUs) in R. Non-metric Multidimensional Scaling (NMDS) was constructed with function *metaMDS* with the *vegan* package, performed with 1000 random iterations and based on Bray-Curtis dissimilarity matrix for the taxonomic relative abundance and the metabolic prediction tables. Permutational multivariate analysis of variance (PERMANOVA) considered surface or incubation mode (nested) as factors with 999 permutations.

Biomarkers were discovered with linear discriminant analysis (LDA) with effect size (LEfSe) (Segata et al., 2011) software using 5% for alpha significant value for Kruskal–Wallis test, and an LDA higher than 2.0. LEfSe software was used to detect discriminant groups between the three surfaces incubated in dynamic mode, as well as between all surfaces incubated in the four incubation modes after 1 year. In the first analysis, surfaces were considered as classes, and all data points were pooled together. For the long-term analysis, incubation modes were analyzed as classes with surfaces as subclasses, as well as the inverse, since both factors were shown to promote microbial community differentiation. As the subclasses were significantly different from each other, the number of biomarkers was greater when the Wilcoxon step was performed by only comparing subclasses within the same class. The analysis was also performed to detect differences between surfaces with incubation modes as subclasses, considering that both factors played a role in discriminating communities.

Function prediction was obtained from the web-based tool MicrobiomeAnalyst (Dhariwal et al., 2017) with the Tax4Fun (Aßhauer et al., 2015) software based on the relative abundance of OTU table (with assigned taxonomy with Silva database 123) for KEGG orthology enzymes, normalized by the 16S rRNA copy number. The functional prediction matrix was matched with a KEGG orthology (KO) table, corresponding KO with subsystems and metabolism (in-house script). Mean abundances of specific subsystems were used for statistical analyses of 2-way ANOVA using surface and mode of incubation as factors.

Graphical representation (*ggplot2*) and statistical analysis were performed with R (version 3.4.2). Differences between time, surface or mode of immersion were tested with one or two-way ANOVA and Tukey HSD *post hoc* test. An unweighted pair group method with arithmetic mean (UPGMA) dendrogram based on Bray-Curtis dissimilarity was used to test grouping between samples with R functions. Beta-diversity was observed by NMDS, and the effects of surface or immersion mode on the communities OTU table were tested with PERMANOVA and ANOSIM, as well as similarity percentage (SIMPER) analysis with 999 permutations *(vegan* package). Further details are described in Supplementary Material.

## Results

### Physicochemical Characteristics of North-Western Mediterranean Coastal Water in Toulon and Banyuls Bays

Water temperature (under 1 m depth) in Toulon Bay varied from 16.9°C (June) to 24.1°C (August), but no significant differences were observed for pH (8.2 ± 0.08) or salinity (38.1 ± 0.07). The low values of dissolved organic carbon (DOC), total nitrogen (TN), and nitrates varied slightly, mainly at the last sampling date and were similar to the mesotrophic characteristics in Banyuls Bay. Banyuls differed from Toulon with lower concentrations of nitrates and trace metals. When compared to the Mediterranean geochemical background, dissolved Zn, Cu, Pb and Cd concentrations in Toulon Bay appeared 32, 16, 13, and 1.9 times higher, respectively (an elemental ratio of 943:260:13:1) (Supplementary Table S1). Those values were 15×, 6×, 6×, and 1.3× higher in Toulon than in Banyuls for Zn, Pb, Cu, and Cd, respectively. Physicochemical parameters were measured only during the first 75 days of each immersion but are consistent with previous descriptions from our group and collaborators (Romero et al., 2014; Misson et al., 2016; Briand et al., 2017; Girard et al., 2017).

### Surface Roughness and Wettability

According to surface characteristics measured before immersion, PVC and the SPC were rougher (highest Ra values) than the FRCs (Supplementary Table S2). However, the waviness showed that the rough PVC surface had more peaks (lower Wa), compared to the more homogenous SPC surface. The FRCs differed in wettability: FRC1 presented a hydrophobic surface (high θstat and θa), with spots of hydrophilicity, as observed with a high hysteresis (H). Due to its amphiphilic copolymer, FRC1 surfaces were expected to have a chemical surface reorganization with different degrees of hydrophilicity depending on the number of hydrophobic and hydrophilic phases (Duong et al., 2015). Despite its inert surface, the PVC presented similar behavior with high hydrophobicity and hysteresis. Note that FRC2 had the most hydrophilic surface.

### Biofilm Density and Alpha-Diversity Over Time Under Dynamic Immersion

**FIGURE 1:**
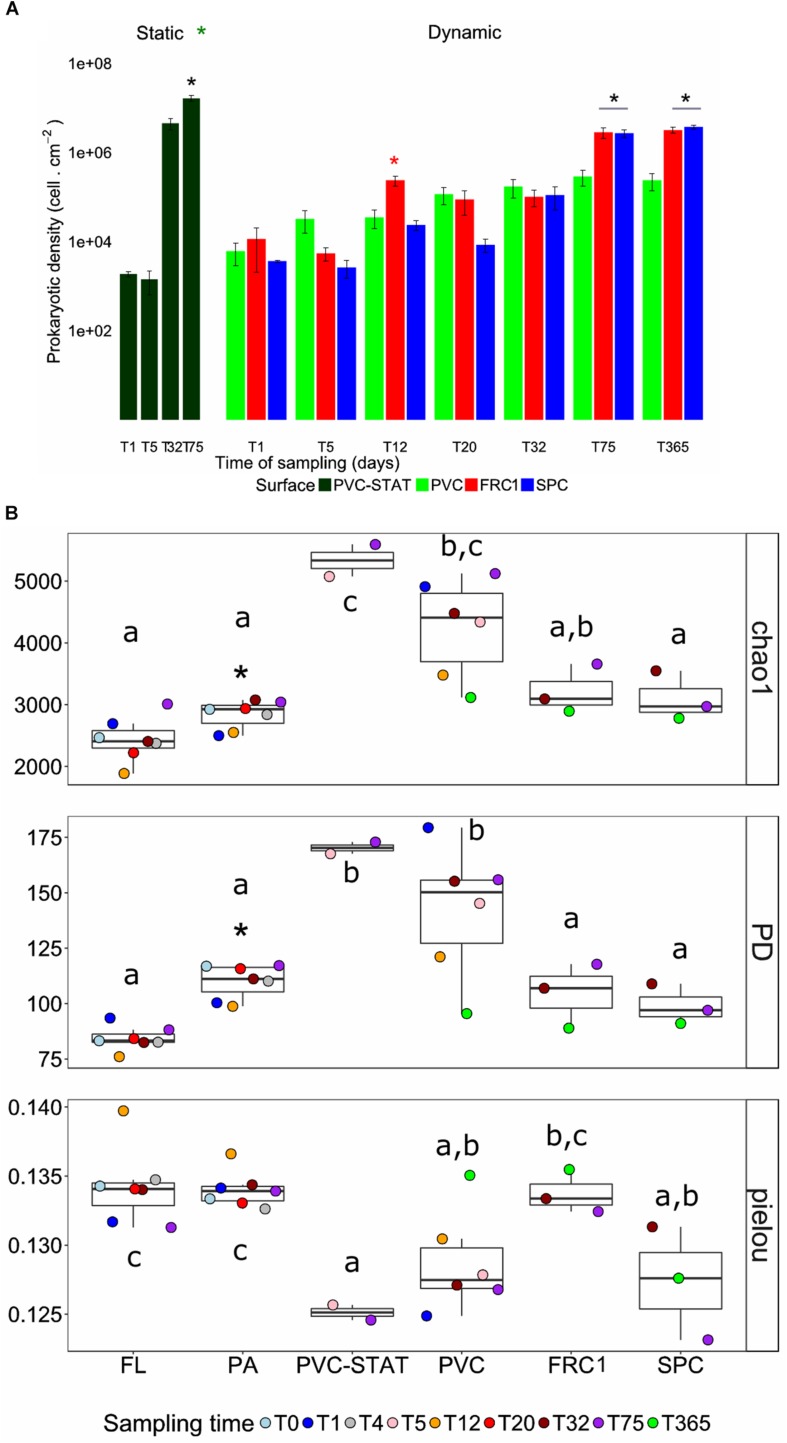
Dynamics of cell density and α-diversity indices over time. **(A)** Bar plots of the density of heterotrophic prokaryotes immersed in dynamic modes on PVC, FRC1, and SPC panels. PVC-STAT represents PVC panels immerged in static mode near the rotor for comparison at 5 and 75 days. **(B)** Chao1, phylogenetic diversity (PD) and Pielou indexes depicted in boxplots representing the distribution of values obtained in different time points (colored accordingly). Whiskers represent the smallest and the largest values. The star represents *t*-test performed between Static and Dynamic modes in cell density, and between PA and FL for alpha-diversity indexes. Letters are according to Tuckey–Kramer *post hoc* tests to compare group means and depict surface significantly different (*p*-value < 0.05) in one-way ANOVA analysis performed per surface over time. PVC, polyvinyl chloride (green); FRC1, fouling release coating (red); SPC, self-polishing coating (blue).

An average of 7 × 10^3^ heterotrophic prokaryotic cells.cm^–2^ were counted over the three types of AF surface from Day 1, and no statistical differences were found between them, except for FRC1 at 12 days (Figure 1A). Over time, the estimated prokaryotic abundance increased significantly, from 2.9 × 10^5^ to 8.4 × 10^6^ cells.cm^–2^ after 75 days (Figure 1A). PVC panels incubated in static mode (PVC-STAT) near the rotor showed initial similar values of prokaryotic cells (1.9 × 10^3^ cells.cm^–2^) with a greater steep rate of colonization after 75 days (1.6 × 10^7^ cells.cm^–2^, Figure 1A). Planktonic cell number was estimated to be around 10^6^ cells.cm^–2^ and did not change over time (Supplementary Figure S2). Biofilm covered less than 0.1% on day one and up to 1.7% after 1 year (Figure 1B).

Chao1, PD and Pielou showed no clear pattern over time for the PVC in dynamic mode, the only substrate from which DNA could be amplified from Day 1, while amplifications from FRC1 and SPC panels were obtained only after 32 days. Higher richness and diversity were observed on PVC in static mode. Distinctively lower richness and diversity on both FRC1 and SPC was noticed (Figure 1B). Only FRC1 showed greater evenness. The fraction of particles attached (PA) planktonic community, present in the 3 μm filter, had higher chao1 and PD than the free living (FL) fraction (0.2 μm filter), but lower than biofilm communities.

### Temporal Variation of Prokaryotic Community Under Hydrodynamic Stress

**FIGURE 2:**
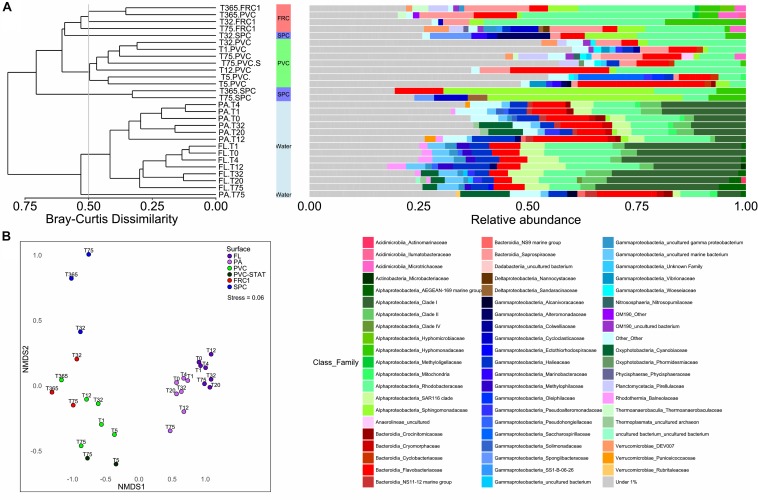
**(A)** Prokaryotic community composition over the immerged surfaces in dynamic mode from 1 day to 1 year. UPGMA clustering formed 6 groups with greater than 50% similarity, portraying the relative abundances of families of Bacteria and Archaea found in each type of surface over time in dynamic mode and in the surrounding water. Families contributing to < 1% were summarized. **(B)** NMDS representation of Bray Curtis dissimilarity between samples according to surface or immersion mode. Stress value under 0.1 denotes good ordination of microbial community samples in two dimensions. PERMANOVA *p*-value was equal to 0.001 in surface effect.

The planktonic community in PA and FL clustered separately (ANOSIM, R 0.7736, *p* = 0.001). Furthermore, prokaryotic communities adhering to the surfaces were significantly different from PA and FL (ANOSIM, R 0.7385, *p* = 0.001) (Figure 2), but the microbial composition of PA resembled biofilm biomarkers (Supplementary Figure S3B). FL had double the relative abundance of SAR11 (Clade I) and SAR116, against the greater abundance of Bacteroidetes, and especially Flavobacteriaceae, Planctomycetes, or Verrucomicrobia, in PA, but similar proportions of Rhodobacteriaceae (Figure 2). Biofilm colonizing PVC incubated in dynamic mode displayed no significant differences with the static mode at the same site (PERMANOVA, *p* > 0.05).

There was little effect of time on the composition of the planktonic communities (PERMANOVA, *p* = 0.5), but a great effect on the communities over surfaces (PERMANOVA *p* = 0.001, ANOSIM-R 0.8739). The PVC surface presented little change in Flavobacteriaceae (from 17% relative abundance after 1 day to 14% after 1 year) but a greater increase in the proportion of Rhodobacteriaceae (from 13% after 1 day to 41% after 1 year) (Figure 2). Despite FRC1 and SPC only being described after 32 days, FRC1 biofilm was similar to PVC composition of Rhodobacteriaceae (ranging from 19 to 44% relative abundance). The SPC, even though mainly colonized by Sphingomonadaceae, was first colonized by Alteromonadaceae (*Glaciecola* spp.) at 32 days (12%) which decreased greatly with time (lower than 1%).

**FIGURE 3:**
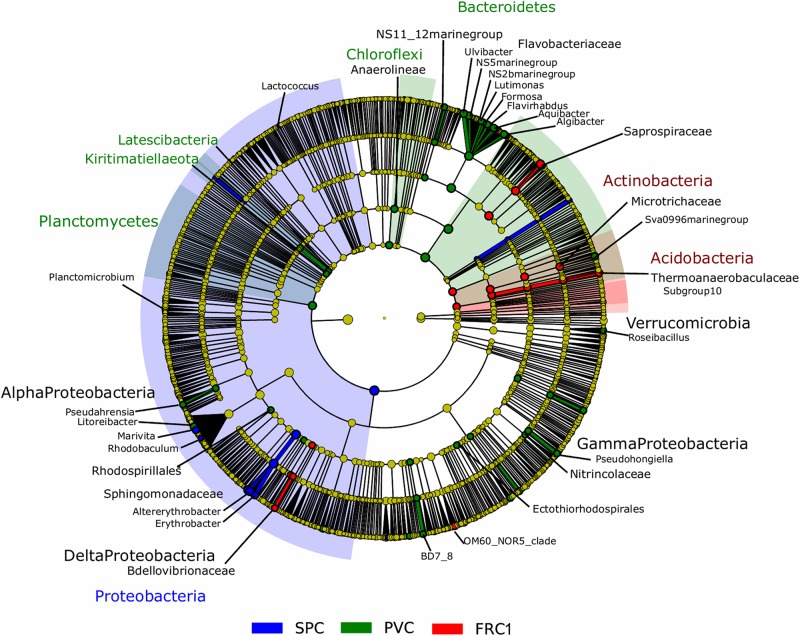
LEfSe analysis cladogram identified discriminant taxa between PVC (green), FRC1 (red), and SPC (blue) biofilm communities. Only taxa with LDA > 2.0 are displayed.

Biofilm composition differed mainly due to the presence of α-Proteobacteria over the SPC; Actinobacteria and Acidobacteria over FRC1; and Bacteroidetes, Planctomycetes, Chloroflexi (and Kiritimatiellaeota and Latescibacteria in lower significance) over the PVC (Figure 3 and Supplementary Figure S4). Sphingomonadaceae was significantly distinct (LDA = 4.3), specifically genera *Altererythrobacter* and *Erythrobacter* (previously classified within Erythrobacteraceae) detected on the SPC panels from 32 days (9% relative abundance) to 1 year (18% relative abundance). Bacteroidetes and Flavobacteriaceae were the most discriminant phylum and family on the PVC panels (LDA = 4.1). Moreover, FRC1 was markedly colonized by Saprospiraceae (values ranging from 3 to 14% relative abundance) from the Bacteroidetes (LDA = 4.0).

Finally, Archaea represented 0.3% on average of the prokaryotic communities in biofilms and 1.1% in planktonic communities, being either Euryarchaeota (unclassified) or Thaumarchaeota (Nitrosopumilaceae). The beta diversity of all prokaryotic communities (Archaea and Bacteria) was highly correlated to only Bacteria beta diversity for all samples (Mantel test *R* = 0.9999, *p* = 0.001).

### Microbial Communities Over Variable Surfaces and Hydrodynamic Pressures After 1-Year

**FIGURE 4:**
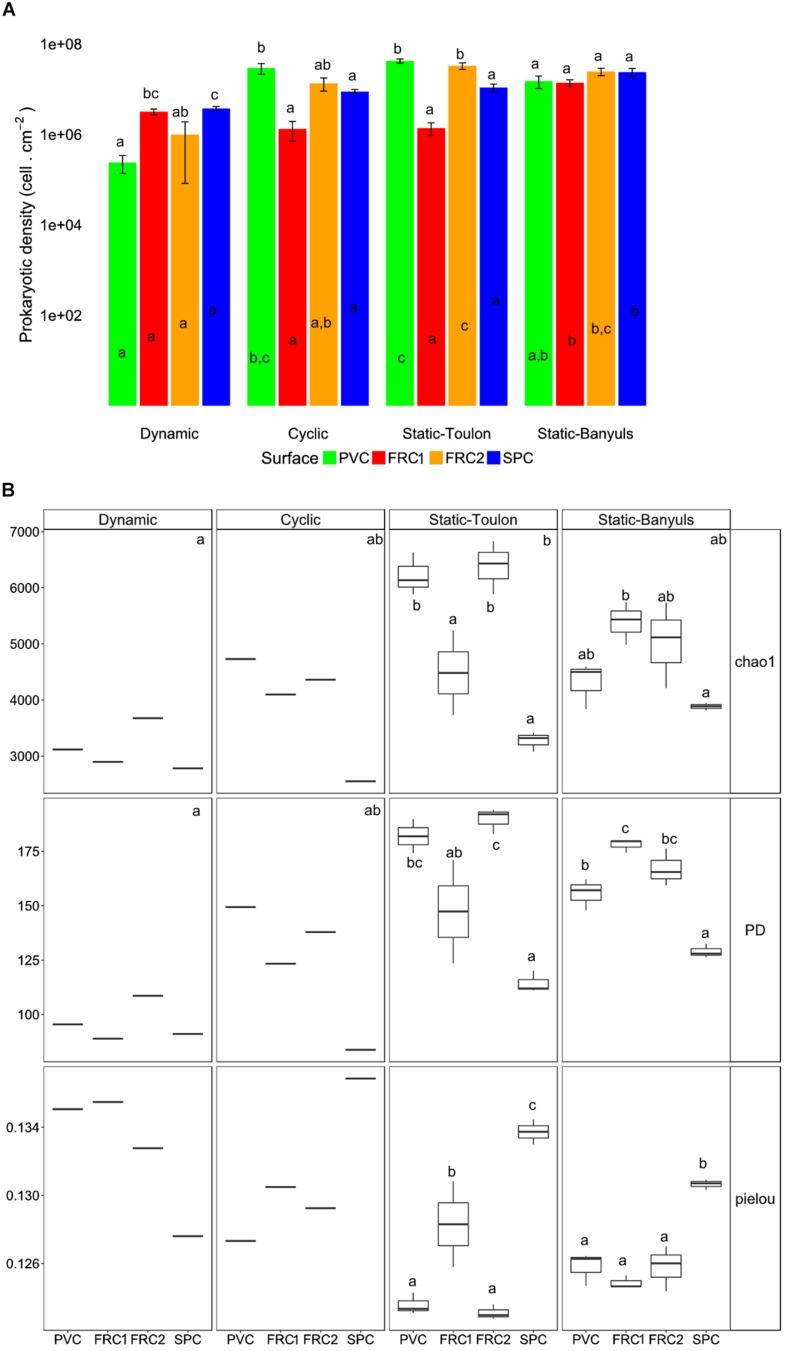
Cell density, surface coverage and α-diversity indexes from biofilms over the four surfaces immersed on the four immersion modes. **(A)** Heterotrophic prokaryotes cell density per cm^2^; **(B)** Chao1, phylogenetic diversity (PD) and Pielou indexes. Whiskers represent the smallest and the largest values. One-way ANOVA analysis were used to detect differences: letters indicate groupings defined with Tuckey’s HSD *post hoc* test (*p*-value < 0.05). Letters inside bars indicate differences between mode of immersion for one surface; letters above bars indicate differences between surfaces within the same mode. Prokaryotic density is directly correlated to surface coverage. In the boxplot, letters in the upper corner of each panel represent difference between immersion modes; letters over boxplots display differences between the mean of surfaces only in the treatments performed in triplicates. PVC, polyvinyl chloride (green); FRC1, fouling release coating (red); FRC2 (orange); SPC, self-polishing coating (blue).

**FIGURE 5:**
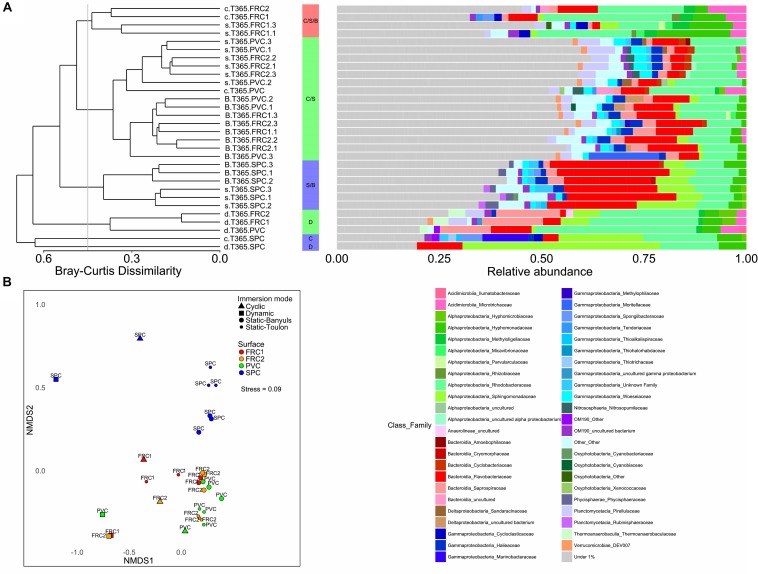
**(A)** Unweighted pair group method with arithmetic mean clustering formed 6 groups with greater than 50% similarity, portraying the relative abundances of families of Bacteria and Archaea found in each type of surface or immersion mode. Families contributing to < 1% were summarized. Clusters were named according with immersion mode, and colored depending on the surface that predominated in the cluster. Cluster formed by PVC, FRC1 and FRC2 was colored as green; only FRC1 as red, and only SPC as blue. Letters represent immersion mode: D as dynamic, C as cyclic, S as static at Toulon and B as Static at Banyuls. **(B)** NMDS representation of Bray Curtis dissimilarity between samples according to surface or immersion mode. Stress value under 0.1 denotes good ordination of microbial community samples in two dimensions. PERMANOVA *p*-value was equal to 0.001 in surface effect and/or immersion mode effect.

Long-term colonization (365 days) was affected by surface and immersion mode (Figures 4, 5). Heterotrophic prokaryote density was the highest on PVC in cyclic and static modes, and lowest in dynamic, with no significant differences between FRC1 and SPC (Figure 4A). The FRCs behaved differently according to site in static mode: FRC2 exhibited higher densities than FRC1 in Toulon, and FRC1 and SPC showed significantly higher densities in static mode at Banyuls compared to Toulon.

Hydrodynamics provided the greatest selection of adhered biofilms in terms of richness and diversity (Figure 4B). Cyclic immersion presented values intermediate between dynamic and static modes. Concerning the type of surface, SPC had lowest richness and diversity in all modes. FRC1 behaved differently in the two sites for static immersion, presenting higher diversity in Banyuls. Furthermore, FRC2 had greatest richness and diversity together with PVC in static immersion in Toulon but lower richness and diversity in Banyuls, showing the interaction effect between site and surface.

Regarding the composition of the taxonomic community, shear stress was the strongest driver for the selection of microbial communities over artificial surfaces (ANOSIM for mode *R* = 0.5, for surface *R* = 0.3), and static immersions were more similar and segregated than cyclic and dynamic immersions (Figure 5 and Supplementary Figure S6). FRC1 and FRC2 clustered together with PVC, but SPC was the most different regardless of the mode (pairwise MANOVA; *p* < 0.05 after false discovery rate adjustment). Furthermore, regarding the sites, the environmental variables explained only 12% (CCA analysis not shown) of the variability observed in the communities in static mode between Toulon and Banyuls, with an average cumulative dissimilarity of 45% (SIMPER analysis, 999 permutations), driven mainly by the dissimilarity of SPC with regard to the other surfaces. However, differences between communities on each surface was greater at Toulon than at Banyuls (Supplementary Table S3).

**FIGURE 6:**
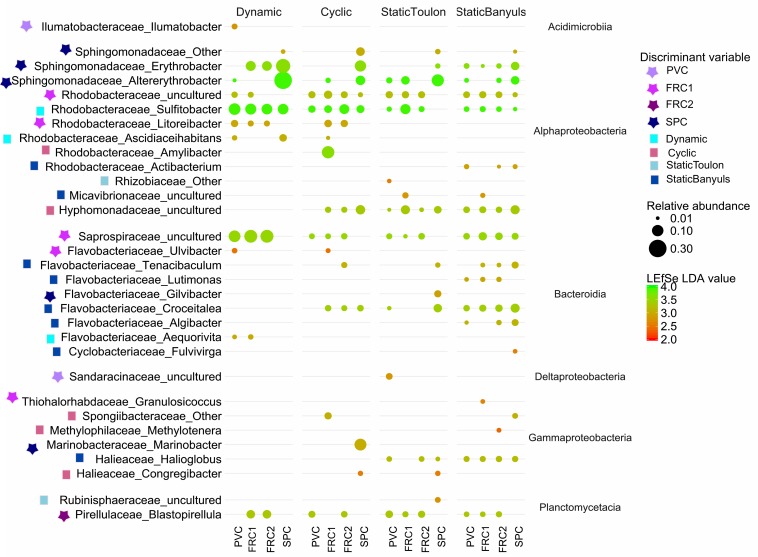
Relative abundance of the discriminant taxa detected by LEfSe in the four types of surface (PVC, FRC1, FRC2, and SPC) over the 4 modes of immersion (dynamic, cyclic, static Toulon, and static Banyuls). Taxonomy is based on SILVA release 132. Taxa lower than 1% are not displayed. Bubble size is proportional to the relative abundance of the genus, and red to green gradient refers to LDA discriminant value. Symbols indicate the discriminant variable.

With greatest abundances and greatest LDA scores (Figure 6), Sphingomonadaceae was discriminative of SPC also after 1 year. Especially in dynamic mode, communities were composed of 29% *Altererythrobacter* compared to 6% in cyclic mode, 3.5% in static-Banyuls and 8.8% in static-Toulon (Figure 6). Likewise, on SPC, *Marinobacter* was discriminated particularly under cyclic mode (11% relative abundance). Saprospiraceae was discriminative for FRC1 especially in dynamic mode (14%), even though this group had similar relative abundance on PVC (10%). Different genera of Rhodobacteriaceae were discriminated according to surface or mode: the genus *Amylibacter* (12%) was particularly abundant on FRC1 under cyclic mode; *Sulfitobacter* was discriminated in dynamic mode; and other Rhodobacteraceae were depicted on FRC1 but present on all samples. Correspondingly, most Flavobacteriaceae genera were discriminant for static immersions, except *Aequorivita*, which was found in dynamic mode.

Network analysis (Supplementary Figure S5) depicted OTUs mostly present on SPC and negatively correlated to many of the OTUs found on other surfaces. This was the case of Flavobacteriaceae *Aquamarina* and Sphingomonadaceae *Altererythrobacter*, both mostly present on SPC, in static-Toulon and cyclic modes, respectively. Likewise, an OTU of the Planctomycetes *Blastopirellula* presented high values of degree and BC, mainly present on PVC and FRC2, and low co-occurrence with Sphingomonadaceae, Leptospiraceae or Porticoccaceae.

### Predicted Functions From Microbial Communities Under All Conditions

**FIGURE 7:**
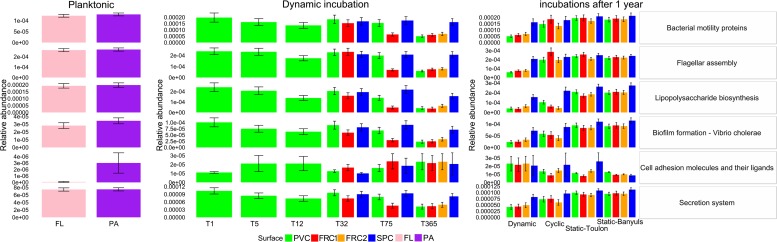
Relative abundance of key subsystems predicted from the microbial community’s present over the different types of surfaces and immersion modes. Key KOs were combined, and their mean abundance presented. Error bars represent standard deviation from the average of KO abundance per subsystem. Only selected subsystems with significant difference are presented. Taxonomy is based on SILVA release 123 for prediction with Tax4Fun, and subsystems are named according to Kegg Orthology hierarchy.

Non-metric multidimensional scaling based on Bray-Curtis dissimilarity from either taxonomic or functional data highlighted that samples are more similar according to function than to taxonomy (Supplementary Figure S6). Considering predicted functions, cyclic and dynamic samples were the most different (almost 30%) after 1 year. Despite high degrees of similarity between samples regarding overall pathways, specific metabolic characteristics were tested separately. These were assumed to be related to cell adhesion or metal tolerance and therefore potentially linked to colonization of the different artificial surfaces (Figure 7). General tendencies potentially related to microbial adherence, were observed as biofilm formation and cell adhesion genes were predicted in significantly greater abundance in biofilm samples. Instead, the planktonic community allowed prediction of greater abundances for flagellar assembly and lipopolysaccharide (LPS) biosynthesis. All selected characteristics followed a similar pattern of greater abundance until 32 days and a significant decrease after 1 year when under dynamic mode. Significantly lower abundances were observed after 1 year in dynamic compared to other modes. Likewise, under shear stress, PVC and SPC presented higher abundances than FRC1, and after 1 year SPC was consistently different from other surfaces. Relative abundances of KOs for cell adhesion molecules were an exception, as the community selected under dynamic mode had greater abundances than the community in static-Banyuls. Consistently greater values obtained for SPC could be related to the slightly greater proportion of taxa used for the prediction (Supplementary Table S4).

## Discussion

In nature, most biofilms are subjected to various degrees of hydrodynamic environments: in marine and river environments, in the human body or in artificial systems such as drinking-water pipes; where the shear stress tends to affect the growing biofilm and its form as reviewed before (Hall-Stoodley et al., 2004). Nevertheless, these studies referred mostly to experimental flow cells under turbulent or laminar flow (Stoodley et al., 1998; Purevdorj et al., 2002; Augspurger et al., 2010) or on stream flumes (Battin et al., 2003). To our knowledge, this is the first study on marine biofilm communities occurring over surfaces under shear stress *in situ* and with 1 year long-term immersion.

### Hydrodynamic Stress Slows Down Assembly of the Microbial Biofilm on Inert Surfaces

PVC, used as reference for marine colonization, was not distinctly more colonized than were surfaces designed to reduce this natural process, and the seemingly lower cell density on PVC when compared to FRC1 and SPC under static mode after 75 and 365 days was possibly a consequence of its greater colonization by bigger organisms (i.e., algae, tubeworms).

Under dynamic immersion, PVC had 10 × fewer cells than in static immersion, and the fold change after 75 days was also affected: 2000- against 20,000-fold increase in static mode (Pollet et al., 2018), interpreted as a colonization delay observed in dynamic conditions when compared to static. This relation between the magnitude of shear stress and biofilm biomass has been observed before in drinking-water systems Tsai (2005). On PVC surfaces, microbial community structure was dominated by Rhodobacteriaceae and Flavobacteriaceae over time. However, although the increase of Rhodobacteriaceae in dynamic conditions occurred from 12 days of immersion, in static mode the appearance of members of this group happened after 4 days (Pollet et al., 2018).

Alternatively, under dynamic conditions, PVC had already presented its greatest richness and diversity after 24 h, dominated by Bacteroidia (Flavobacteriaceae and Saprospiraceae). While Saccharospiraceae (new nomenclature for Oceanospirillaceae in Silva132) and Alteromonadaceae were considered as pioneers in PVC biofilm in static mode (Pollet et al., 2018), the greatest abundances were observed after 5 days under shear stress and were approximately 10-fold less abundant than in static. Therefore, hydrodynamics influenced the time of development and the selection of different groups over PVC.

Besides immersion mode, sampling sites showed lower but significant (PERMANOVA, *p* < 0.001) differences in microbial community structure. Despite the general oligo- to mesotrophic characteristics of the Mediterranean Sea and similar physicochemical conditions (Lambert et al., 2018), Toulon Bay is considered more polluted by trace metallic elements and contains slightly higher nutrient concentrations when compared to Banyuls (Coclet et al., 2019). Such environmental differences could have resulted in dissimilar community shaping on inert surfaces, as previously reported (Webster and Negri, 2006; Briand et al., 2017). However, in accordance with the conclusions on dynamic vs. static mode at Toulon in this study, it appears that higher shear stress resulting from the natural hydrodynamic conditions under the SOLA buoy at Banyuls acted as the main driver for surface colonization in terms of microbial community density and structure, limiting the effect of surface characteristics. Although the hydrodynamic force applied by the current was not measured, we assumed that the surfaces in Toulon were more protected in the enclosed harbor. In fact, the immersion at Banyuls Bay was more prone to wave movement and possibly subjected to greater hydrodynamics.

Considering that immersion lasted 1 month in previous studies (Webster and Negri, 2006; Briand et al., 2017) compared to 1 year in this case, time could have exaggerated this phenomenon. As discussed before, in laboratory incubations a core community predominates when subjecting two different planktonic communities to shear stress (Niederdorfer et al., 2016), as hydrodynamics is a stronger driver of microbial community structure in biofilms than is source community.

The rotor used for dynamic immersions rotated at 15 knots, equivalent to 7 m s^–1^, which is 10 times faster than tests reported in the literature, where 60 cm s^–1^ was the threshold for flow velocity on biofilm growth (Tsai, 2005). Whatever the substrate, hydrodynamics affected the microbial density and diversity. Even after 1 year, dynamic shear stress negatively affected the biofilm diversity, especially on SPC, slowing down biofilm maturation and preventing microbial communities reaching the structure observed in static mode.

As in natural marine ecosystems surfaces are not only submitted to either static or dynamic conditions, we created a cyclic mode to test the speed gradient between dynamic, cyclic and static modes. Successive transitions between both static and dynamic conditions had little effect on cell densities over the year. Instead, richness and diversity values were intermediate to dynamic and static modes for all surfaces, with the lowest found onSPC.

Despite having different community structure, biofilms observed on the different surfaces with the same shear stress presented similar cell density. However, we do not know the biofilm topography as observed by the Non-destructive technique of biofilms imaging *in situ* (Fischer et al., 2014).

Biofilms grown under flow present modified exopolymeric substances (EPS) to withstand shear stress, varying with the microbial diversity (Hall-Stoodley et al., 2004) and associated with the strength of adhesion (Dang and Lovell, 2016). Under high shear conditions, biofilms can grow smoother and therefore be less susceptible to the force (Nam et al., 2000). Future analysis should test how marine biofilms are affected in terms of elasticity and thickness under turbulent flow (Herbert-Guillou, 2001).

Surfaces behaved differently according to immersion mode, and the shear stress effect was predominant in PVC and FRC2 cell densities. This effect of hydrodynamics decreasing biofilm diversity and maturation has already been observed in laboratory incubations (Rochex et al., 2008; Thomen et al., 2017), and is congruent with the observed effect of hydrodynamics on initial microbial community colonization (Thomen et al., 2017). Despite the advantages of this *in situ* approach, it did not enable assessment of the equilibrium between recruitment and detachment occurring in the biofilms, and it is possible that lower microbial biomass observed under dynamic mode resulted from higher rates of detachment, as observed with increasing shear stress in laboratory experiments (Tsai, 2005). In consequence, it remains to be tested if whether biofilm colonization is more random, or less deterministic, under shear stress. Alternatively, hydrodynamic forces could select organisms with different cell surface charge and composition that allow for greater adhesion.

### Surface and Mode of Immersion Interact in Selecting Microbial Communities

Microbial diversity in marine biofilms differed from the surrounding water, as previously described (DeLong et al., 1993; Ghiglione et al., 2007; Crespo et al., 2013). Moreover, biofilms showed greater richness and diversity than PA, which was in turn more diverse than FL, in accordance with data from the NW Mediterranean Sea (Crespo et al., 2013) and the brackish Baltic Sea (Rieck et al., 2015). FL had distinctly more α- (namely SAR11 and SAR116) and γ-Proteobacteria, as well as more Thaumarchaeota. Despite being more similar to the microbial structure of FL (Ghiglione et al., 2007), PA discriminant groups such as Bacteroidetes, Planctomycetes and Verrucomicrobia resembled the biofilm communities on PVC, which is coherent with the concept of particle attachment (Grossart, 2010).

A core biofilm present on all four surfaces independent of immersion mode was revealed in the co-occurrence network, and OTUs with highest centrality were assigned to Rhodobacteriaceae and Flavobacteriaceae (15 and 14.6% of OTUs, respectively). A predominance of α-Proteobacteria, Bacteroidetes and γ-Proteobacteria has been described for biofilms from the Atlantic and Pacific Oceans (Dang and Lovell, 2016). However, no study has described the relative impact of location, time, surface and mode of immersion together. Lower-rank variations occurred depending on these factors. Within the α-Proteobacteria, Rhodobacteriaceae is often found in marine biofilms (Elifantz et al., 2013), but different genera can be discriminated, such as, for example, *Roseobacter* on glass (Elifantz et al., 2013). Here, variation according to immersion mode discriminated *Sulfitobacter* or *Ascidiaceihabitans* in dynamic mode and *Amylibacter* in cyclic mode. This reinforces the ecological coherence (Philippot et al., 2010) of Rhodobacteriaceae in the formation of aquatic biofilms, but also the specificity in lower taxonomy ranks depending on the conditions tested. The network depicted the impact of hydrodynamics and biocides in the selection of organisms, as the four nodes with greatest degree of negative correlations were those of *Altererythrobacter, Aquamarina*, and *Porticoccus* mainly present on SPC surfaces, and of *Blastopirellula* in dynamic immersion (PVC, FRC1, and FRC2). However, the lack of replicates in both dynamic and cyclic modes calls for caution in the interpretation of certain group abundance, and this finding should be looked on as preliminary data for further hypotheses.

Similarly, Proteobacteria were variable according to the surface, as α-Proteobacteria seemed more capable developing over SPC, γ-Proteobacteria on PVC and a few δ-Proteobacteria were specifically discriminant on FRC1. Similarly, Bacteroidetes, commonly described in marine biofilms (Salta et al., 2013), mainly composed of Flavobacteriaceae, were present on all four surfaces. Under dynamic mode, several genera of Flavobacteriaceae were discriminant on PVC surfaces, a keystone group previously studied under static mode (Pollet et al., 2018). However, the Saprospiraceae, another Bacteroidetes family, was detected on FRC1. This has been associated with the use of complex carbon sources (Xia et al., 2008; Oberbeckmann et al., 2016) and often described on the plastisphere (Oberbeckmann et al., 2016, 2018), but also on the surface of marine eukaryotes, such as the Ulvacean algae (Tujula et al., 2010). Saprospiraceae were also discriminated on FRC1 particularly under dynamic mode, despite the poly (ethylene glycol; PEG) property that hampers cell adhesion on FRC1 (Galli and Martinelli, 2017). Their ability to colonize elastic surfaces has been also shown in the coast of China (Yang et al., 2016), and could be related to the formation of streamers (Besemer et al., 2009) due to their filamentous morphology.

The new generation of fouling release surfaces do not have biocides in their composition, but select organisms based on physical constraints. For bacterial biofilms, the effect of surface wettability on biofilm formation (Dang and Lovell, 2000) coincides with lower surface energy (Lobelle and Cunliffe, 2011). Although effective for most macrofoulers (Lejars et al., 2012), which are removed by hydrodynamic stress, few differences were observed in the decrease in microbial cell abundance, not even under dynamic or cyclic modes. Furthermore, PVC, SPC and the hybrid FRC2 could be classified as harder surfaces in comparison to FRC1, which is a soft surface (Lejars et al., 2012). Besides, the hydrophilic surface of FRC2 behaved more similarly to the hydrophobic but rough surface of PVC than to the amphiphilic surface of FRC1 (Duong et al., 2015). As observed in medical devices, hydrophobicity and roughness are positively correlated with bacterial adhesion (Sousa et al., 2009).

### Strong Prokaryotic Selection in Biocidal Artificial Surfaces

Regardless of the immersion mode, the SPC was more than 60% dissimilar than to other surfaces. This was mainly due to Sphingomonadaceae, and particularly the genera *Altererythrobacter* and *Erythrobacter*, despite their being found in the surrounding metal-contaminated waters in very low abundance (less than 0.5% and around 2% of abundance in FL and PA fractions, respectively). Lower prokaryotic diversity was congruent with the effect of metal biocides on bacterial diversity (Muthukrishnan et al., 2014; Briand et al., 2017). The influence of surfaces containing copper has indeed already been identified on some bacterial groups such as Alteromonadaceae in Toulon Bay (Briand et al., 2017) and Flavobacteriia in tropical Oman seawater (Muthukrishnan et al., 2014). However, Alteromonadaceae, and especially *Alteromonas* spp., seemed to be selected earlier in time, whereas *Erythrobacter* appeared progressively after 75 days under static mode in the bay of Toulon (Pollet et al., 2018). Similarly, Sphingomonadaceae was correlated with copper tolerance in copper-contaminated groundwater (Vilchez et al., 2007). Sphingomonadaceae, Alteromonadaceae, and Oceanospirillaceae are all families that seem to be especially suited to developing biofilms over artificial surfaces in the marine environment, and have been previously described in marine biofilms on surfaces exposed to triclosan (Eriksson et al., 2018).

Furthermore, Sphingomonadaceae were distinctly described on SPC under shear stress. Higher shear can lead to smoother and condensed biofilms, where the ratio between biofilm surface and volume did not differ with shear stress (Nam et al., 2000). Flow velocity can destroy formed biofilm structure (Tsai, 2005), but also increase adhesion force, as shown for *Sphingomonas wittichii* in reverse osmosis membranes due mainly to the presence of glycosphingolipids instead of lipopolysaccharides in the external membrane, and to its EPS.

Assuming that biofilms are more resistant than their planktonic counterparts to a wide range of biocides (Hoyle and Costerton, 1991), the SPC surface could still be toxic to most organisms after 1 year, due to the renewal of the surface by erosion over time under cyclic and dynamic conditions (data not shown). Biofilms can protect cells within by EPS protection (Le Costaouëc et al., 2012) or by metals immobilization to detoxify the cell. While this metal-ion biosorption depends on the organism and on the environment (Harrison et al., 2007), Sphingomonadaceae seem to use this mechanism as bioaccumulation of copper complexed to phosphates and silicates and embedded in the EPS matrix (Vilchez et al., 2007). The increase in Sphingomonadaceae abundance over time, composing half of the community over the SPC under dynamic mode, seems coherent with findings of *Sphingomonas* spp. in water treatment and supply plants under flow. On the other hand, the predominance of *Altererythrobacter* (Kwon et al., 2007; Xue et al., 2012) was shown to influence negatively the co-occurrence with OTUs from many other groups such as Cyanobacteria, Flavobacteriaceae, Saprospiraceae, Pirellulaceae, Rhodobacteriaceae, but also some γ- or δ-Proteobacteria (Bradymonadales and Myxococcales).

### Looking for Traits Related to Microbial Selection

The dichotomy between free-living and benthic microorganisms matches their lifestyles (Favre et al., 2018). Therefore, niche selection in terms of function should reflect microbial communities. Pioneer biofilm microbial communities can have structures around 25% similar between sites and types of substrata (Briand et al., 2012), suggesting there is a common selection of taxa that are able to colonize, and/or opportunists that later become part of the biofilm. It is also known that multiple phylogenetic groups can perform the same function, which has been known as functional redundancy (Louca et al., 2018). Most recently, a correlation between taxonomic and functional data despite the change in microbial groups with time, brought into question the relevance of functional redundancy in marine microorganisms and highlighted the many functions still unknown (Galand et al., 2018). Here, we assumed that the greater similarity observed between samples in terms of function rather than taxonomy was due to a mixture of factors: redundancy, ecological coherence between taxa in higher ranks (Philippot et al., 2010) and limited database information. Regarding the latter, the fraction of taxonomic units explained (FTU) metric indicated that function prediction was obtained from 65% of the community on average. Notwithstanding the bias inherent in functional prediction from taxonomic data, general trends regarding adhesion to the surface over time, and greater in modes with lesser shear stress, were observed.

Cell adhesion depends on the surface hydrophobicity, roughness and chemistry. Furthermore, adhesion is facilitated by cell motility, the presence of flagella or pili, or the presence of an outer membrane and the LPS in the gram negative bacteria (Gutman et al., 2014). We found that estimated KOs related to motility (Chagnot et al., 2013) (motility proteins, flagellar assembly) varied over time, between surfaces and between immersion modes. Facilitation of bacterial colonization by motility (Kiorboe et al., 2002) did not seem to be related to the selection of organisms adhering to any surface under dynamic mode. Nor was it increased in the communities colonizing the soft amphiphilic FRC1 surface, as flagellated organisms, with their greater ability to adhere, would be expected to have greater abundance (Bruzaud et al., 2015).

Despite the predominance of Non-specific mechanisms being involved in adhesion to abiotic surfaces (Dunne, 2002), the use of copper in paint showed significant effects in discriminating the community due to metal toxicity. We wondered about their genomic abilities to tolerate metal contamination, as observed in uranium-tolerant *Microbacterium* strains with increased transporters in the proteome as an efflux system for trace metallic elements (Gallois et al., 2018). Therefore, secretion systems in higher abundance in SPC communities could be related to the resistance to copper oxide and pyrithione.

## Conclusion

Marine biofilms are known to have relevant ecological functions such as, for example, biogeochemical cycling and the influence on macroorganism settlement. However, they are often studied only in protected areas with little hydrodynamic influence, and not considering the intrinsic contribution of flow in marine conditions. This is the first study to evaluate this effect of hydrodynamics on the selection of biofilm microbials. Our findings suggest contrasting communities at specific phylogenetic levels according to shear stress and surface, despite a core community in high rank level. Hydrodynamics appeared to slow down biofilm growth and disturb structuration. Moreover, hydrodynamics is proposed to partially mask surface properties with time. Microbial discrimination can have an effect either in fundamental studies to understand oceanic hydrodynamics on biofilm turnover, as well as in the applied science of the colonization of microfouling on ships. Furthermore, with an increasing trace metal contamination of seas, related possibly to the fleet of ships worldwide, we show the specialization of Sphingomonadaceae to colonize biocides-containing surfaces.

## Data Availability

The datasets generated for this study can be found in NCBI SRA database, Bioproject PRJNA504753

## Author Contributions

EC analyzed all the data and wrote the manuscript. TP, RB-M, and J-FB achieved the experiments including all immersions and sampling. MM and CB were responsible for surface’s characterization. BM and CG performed water physicochemical and flow cytometry analyses, respectively. J-FG was responsible for surface’s immersion in Banyuls-sur-Mer. J-FB coordinated the study. All authors contributed to the final editing and approval of the manuscript.

## Conflict of Interest Statement

The authors declare that the research was conducted in the absence of any commercial or financial relationships that could be construed as a potential conflict of interest.
