# Supplementary material for: Shear Stress as a Major Driver of Marine Biofilm Communities in the NW Mediterranean Sea
Source: Front Microbiol. 2019 Jul 31;10:1768. doi: 10.3389/fmicb.2019.01768 (PMC6774042; doi:10.3389/fmicb.2019.01768)
Supplement: Supplementary file 1 [file Data_Sheet_1.pdf]

Supporting Information

for

Shear stress as a major driver of marine biofilm communities in the NW Mediterranean Sea

E. C. P. CATÃO [1*], T. POLLET [1,2], B. MISSON [3], C. GARNIER [3], J-F. GHIGLIONE[4], R. BARRY-MARTINET [1], M. MAINTENAY [1], C. BRESSY [1], J-F. BRIAND [1*]

[1] Université de Toulon, Laboratoire MAPIEM, EA 4323, 83041 Toulon, France

[2] UMR BIPAR, INRA, ANSES, ENVA, Université Paris-Est, Maisons-Alfort, France

[3] Univ Toulon, Aix Marseille Univ., CNRS/INSU, IRD, MIO UM 110, Mediterranean Institute of Oceanography, La Garde, France

[4] CNRS, UPMC, UMR 7621, Laboratoire d'Océanographie Microbienne, Banyuls sur Mer, France

*Corresponding authors : elisa.catao@univ-tln.fr, briand@univ-tln.fr

Laboratoire MAPIEM, E.A.4323, SeaTech Ecole d'Ingénieur, Université de Toulon, CS 60584, 83041 Toulon Cedex 9, France

Material and Methods

Experimental design and sampling

Pressure forces were considered negligible face to the rotation speed as a uniform annular flow with tangential force along the panels, due to few impacts of waves in static barge, and higher speed from rotor than waves.

During dynamic incubation, water samples were collected for determination of environmental parameters. Water, temperature, pH and salinity were measured using a Hydrolab® DS5X probe (Hatch Hydromet, USA). Dissolved organic carbon (DOC) and total nitrogen (TN) were analyzed on a TOC-VCSH analyzer (Shimadzu) (Oursel et al., 2013). Nutrients (NO3-, PO43-, Si(OH)4) were analyzed using standard colorimetric methods for seawater (Coclet et al., 2018). Trace metals (Cd, Cu, Pb, Zn), were determined by voltammetry on Metrohm/Ecochemie system (Cindrić et al., 2015). A geochemical signal for the Mediterranean Sea was calculated based on the literature (Morley et al., 1997; Tankere and Statham, 1996), leading to averaged values of 0.054 nM for Cd, 1.45 nM for Cu, 0.096 nM for Pb and 2.7 nM for Zn, and an elemental ratio of 50:27:1.8:1 for Zn:Cu:Pb:Cd.

Surface parameters were determined before immersion. Wettability was evaluated by measuring the static water contact angle value θ_{stat} using a contact angle meter DIGIDROP (GBX Instrument). Each measurement was obtained after cleaning the surfaces with deionized water, five droplets of 1 µL-deionized water were deposited, and the mean water contact angle value was given. Dynamic contact angle measurements experiments were carried out by the advancing-receding drop method under ambient conditions. The deionized water drop is grown on a syringe tip and is picked up by the surface with a withdrawal rate of 0.75 $\mu L.s^{-1}$ and the final droplet volume is 5-10 µL. The syringe tip never leaves the liquid drop. For each coating, the reported advancing (θ_a) and receding (θ_r) water contact angles were the average values obtained from 1 cycle of advancing-receding on 5 deionized water droplets. The hysteresis H ($H=\theta_a-\theta_r$) was also calculated.

Surface roughness profiles were measured by a contact type stylus profiler (Taylor Hobson) using a 2 µm radius tip and a 0.1 µm radius diamond tip with a minimum applicable 1 mN stylus load. The stylus moved across 15 mm length of the coating surface at a constant velocity of 0.50 mm/s to obtain surface height variation. Ra values were assessed from the average of three measurements. According to ISO 4288-1996, the selected cut-off was λc = 0.8mm since Ra < 2 µm. The λs value of 0.0025 mm was taken for all samples.

Sequence data analysis

Raw sequences were merged with *join_paired_end* with QIIME (version 1.9.1) (Caporaso et al., 2010) (default parameters), quality filtered (phred > 29), dereplicated, and passed by chimera checking (usearch61). Primers were trimmed and reassignment of sequences names (to keep track according to sample) was performed with Prinseq-lite (Schmieder and Edwards, 2011). *Screen.seqs* from mothur (Schloss et al., 2009) was used to clean sequences for homopolymers and size filtering (200 and 411 bp; maximum size estimated from primers amplicon region). OTU clustering was performed with uclust_ref (clustering against database first and secondly within own data if no reference is found) at 97% similarity threshold against SILVA release 132 with taxonomic assignment over 90% confidence threshold. Singletons and chloroplasts-identified OTUs were excluded from further analysis and libraries were rarefied to the smallest library size

(10850 reads per sample). Relative abundance was calculated to the total of assigned OTUs. One sample (FRC1 from static Banyuls) was excluded due to low (<2000) read counts.

Network co-occurrence analysis was performed with CONET plugin (Faust and Raes, 2016) within Cytoscape version 3.6.1 (Shannon et al., 2003; Smoot et al., 2011) based on the OTU table of the all surfaces (PVC, FRC1, FRC2 and SPC) in all incubation modes after 1 year incubation, and following the tutorial available in CoNet website (http://psbweb05.psb.ugent.be/conet/tutorial4/php) with slight modifications. Significant correlations (positive and negative) from OTUs with 16 (presence in half of samples) as minimum row occurrence were obtained by a combination of Spearman's correlation and Bray-Curtis or Kullback-Leibler dissimilarities, selecting the 1000 top and bottom edges in the automatic threshold. CoNet was used for rows shuffling and detection of only correlations with p-value < 0.5 after Benjamini-Hochberg multiple test correction and bootstrap filtering of unstable edges (Brown method for p-value merge). MCODE (Bader and Hogue, 2003) was further used to detect clusters present within the whole network, with default parameters except the use of a more restrictive K-core of 3 to filter smaller clusters. The links between taxon from different levels was not explored, to obtain only the effect at the OTU level. Treating the network as undirected showed a clustering coefficient (CC) of 0.269 and heterogeneity of 1.562. We selected the OTUs with the top 25% values of degree (7 to 39) and BC (0.005 to 1).

Table S1. Physicochemical characteristics at the site for the dynamic incubation from 0 to 75 days (average). T: temperature; DOC: dissolved organic carbon; TN: total nitrogen; NO_3: nitrates; PO_4^{-2}: phosphates; $Si(OH)_4$: silicates; Zn: zinc; Pb: lead; Cd: cadmium; Cu: copper. Trace metal (TM) are presented in dissolved concentrations. Banyuls data were obtained with the SOMLIT survey except for the TM.

		**Temperature** (°C)	**pH**	**Salinity**	**Zn** (nM)	**Cd** (nM)	**Pb** (nM)	**Cu** (nM)	**DOC** (mg C .L^{-1})	**TN** (µM–N)	**$Si(OH)_4$** (µM-Si)	**PO_4^{3-}** (µM P)	**NO_3^-** (µM N)
**Dynamic - Toulon**	**T0**	18	8.24	38.1	106.8	0.10	1.1	33.6	1.0	5.8	0.55	undetected	0.42
	**T1**	17.7	8.31	38	96.6	0.12	1.2	23.5	0.97	4.9	0.42	0.02	0.25
	**T5**	17.7	8.59	38.1	71.2	0.09	1.8	16.8	1	5.4	0.64	0.02	0.021
	**T12**	19.5	8.06	37.9	62.4	0.11	0.8	10.6	0.94	5.0	0.82	0.12	0.42
	**T32**	16.9	8.05	38.1	66.2	0.07	0.9	17.8	0.97	5.2	0.52	0.05	0.51
	**T75**	24.1	8.18	38.4	117.1	0.11	2.0	42.1	1.2	8.5	1.1	0.09	0.62
**Static - Toulon**	**T0**	23.4	8.13	37.1	173	0.23	1.8	62.9	1.34	10.7	4.91	0.13	1.67
	**T1**	23.3	8.18	37.6	187	0.15	1.8	49.7	1.27	5.9	2.29	0.00	0.79
	**T4**	22.8	8.09	36.8	289	0.19	2.3	78.5	1.39	12.7	5.1	0.14	1.98
	**T8**	22.3	8.07	37.9	308	0.14	2.2	62.3	1.22	9.2	3.16	0.06	1.15
	**T12**	23.5	7.99	38.1	318	0.14	2.7	63.2	1.37	14.4	1.85	0.14	1.41
	**T20**	25.8	7.98	38.2	290	0.15	2.7	59.9	1.37	15.3	2.03	0.06	0.89
	**T28**	25.2	8.01	38.1	347	0.18	1.4	24.2	1.22	10.5	1.88	0.05	0.58
	**T75**	23.4	8.07	38.5	395	0.21	3.2	39.3	1.44	12.9	2.27	0.03	0.84
**Banyuls**	**T4**	-	8.16	-	6.0	0.08	0.14	4.1	1.23	17.4	0.31	0.02	0.02
	**T18**	-	8.14	-	-	-	-	-	-	-	0.79	0.02	0.02
	**T32**	22.8	8.14	37.8	-	-	-	-	-	-	0.56	0.01	0.02
	**T53**	22.3	8.10	37.9	-	-	-	-	-	-	0.51	0.01	0.02
	**T74**	20.8	8.07	37.9	5.7	0.07	0.31	4.2	0.94	5.7	0.81	0.01	0.04

Table S2. Physical and chemical surface parameters measured before immersion: roughness, waviness and wettability. Ra and Wa: arithmetic mean deviation; Rz and Wz: average based on the five highest and lowest peaks and valleys, respectively; Rsm and Wsm: calculated from the root mean squared. θa: advancing water contact angle; θr: receding water contact angle; H: hysteresis of contact angle; θstat: static water contact angle.

Surface parameters		PVC	FRC1	FRC2	SPC
Roughness	Ra (μm)	2.2±0.3	0.4±0.1	0.7±0.2	1.4±0.1
	Rz (μm)	22.5±2.8	2.1±0.6	5.5±1.9	8.1±0.9
	Rsm (μm)	161±20	447±15	458±169	137±24
Waviness	Wa (μm)	0.5±0.2	3.2±1.4	1.9±0.9	2.1±0.9
	Wz (μm)	3.5±0.8	4.4±1.8	3.6±1.1	3.7±0.9
	Wsm (μm)	4430±921	2967±664	2699±634	2101±645
Wettability	θa (°)	100±8	105±1	77±5	83±4
	θr (°)	69±6	71±4	28±8	75±3
	H	30±7	34±4	49±6	7±5
	θstat (°)	96±9	106±2	80±6	96±9

Table S3. Pairwise comparisons of overall contribution for dissimilarities between communities on each surface immersed in static mode per site. Simper analysis, 999 permutations.

	**Toulon**	**Banyuls**
**PVC-FRC**	48%	26%
**PVC-FRC2**	23%	25%
**PVC-SPC**	61%	47%
**FRC-SPC**	62%	43%
**FRC2-SPC**	60%	41%
**FRC-FRC2**	47%	22%

Table S4. Fraction of taxonomic units (1-FTU) used for the functional prediction per sample and according to incubation mode and comparison performed.

Comparison	Incubation mode	Sample	1-FTU
Long-term incubation (365 days)	Static Banyuls	B.T1an.FRC1.1	0.5967
		B.T1an.FRC1.2	0.6349
		B.T1an.FRC1.3	0.6076
		B.T1an.FRC2.1	0.6072
		B.T1an.FRC2.2	0.6284

		B.T1an.FRC2.3	0.6271
		B.T1an.PVC.1	0.6209
		B.T1an.PVC.2	0.5774
		B.T1an.PVC.3	0.7027
		B.T1an.SPC.1	0.7026
		B.T1an.SPC.2	0.693
		B.T1an.SPC.3	0.7137
		c.T1an.FRC1	0.6489
	Cyclic	c.T1an.FRC2	0.5892
		c.T1an.PVC	0.6591
		c.T1an.SPC	0.7744
		s.T1an.FRC1.1	0.5858
		s.T1an.FRC1.3	0.5648
		s.T1an.FRC2.1	0.5743
		s.T1an.FRC2.2	0.61
		s.T1an.FRC2.3	0.5656
	Static Toulon	s.T1an.PVC.1	0.5936
		s.T1an.PVC.2	0.6229
		s.T1an.PVC.3	0.6406
		s.T1an.SPC.1	0.6709
		s.T1an.SPC.2	0.6743
		s.T1an.SPC.3	0.6977
		d.T1an.FRC1	0.6786
	Dynamic	d.T1an.FRC2	0.6723
		d.T1an.PVC	0.6402
		d.T1an.SPC	0.8534
		d.T1.PVC	0.6142
		d.T12.PVC	0.7695
		d.T32.FRC	0.5317
		d.T32.PVC	0.5933
		d.T32.SPC	0.7167
	Dynamic	d.T5.PVC	0.7612
		d.T5.PVC.STAT	0.6643
		d.T75.FRC	0.5423
Incubation under shear stress over time		d.T75.PVC	0.5311
		d.T75.PVC.STAT	0.6552
		d.T75.SPC	0.7387
		d.02micro.T0	0.6863
		d.02micro.T1	0.6854
	FL	d.02micro.T12	0.6702
		d.02micro.T20	0.5547
		d.02micro.T32	0.5774
		d.02micro.T4	0.6593

	d.02micro.T75	0.6292
	d.3micro.T0	0.6538
	d.3micro.T1	0.6234
	d.3micro.T12	0.7187
PA	d.3micro.T20	0.6109
	d.3micro.T32	0.612
	d.3micro.T4	0.5907
	d.3micro.T75	0.6348

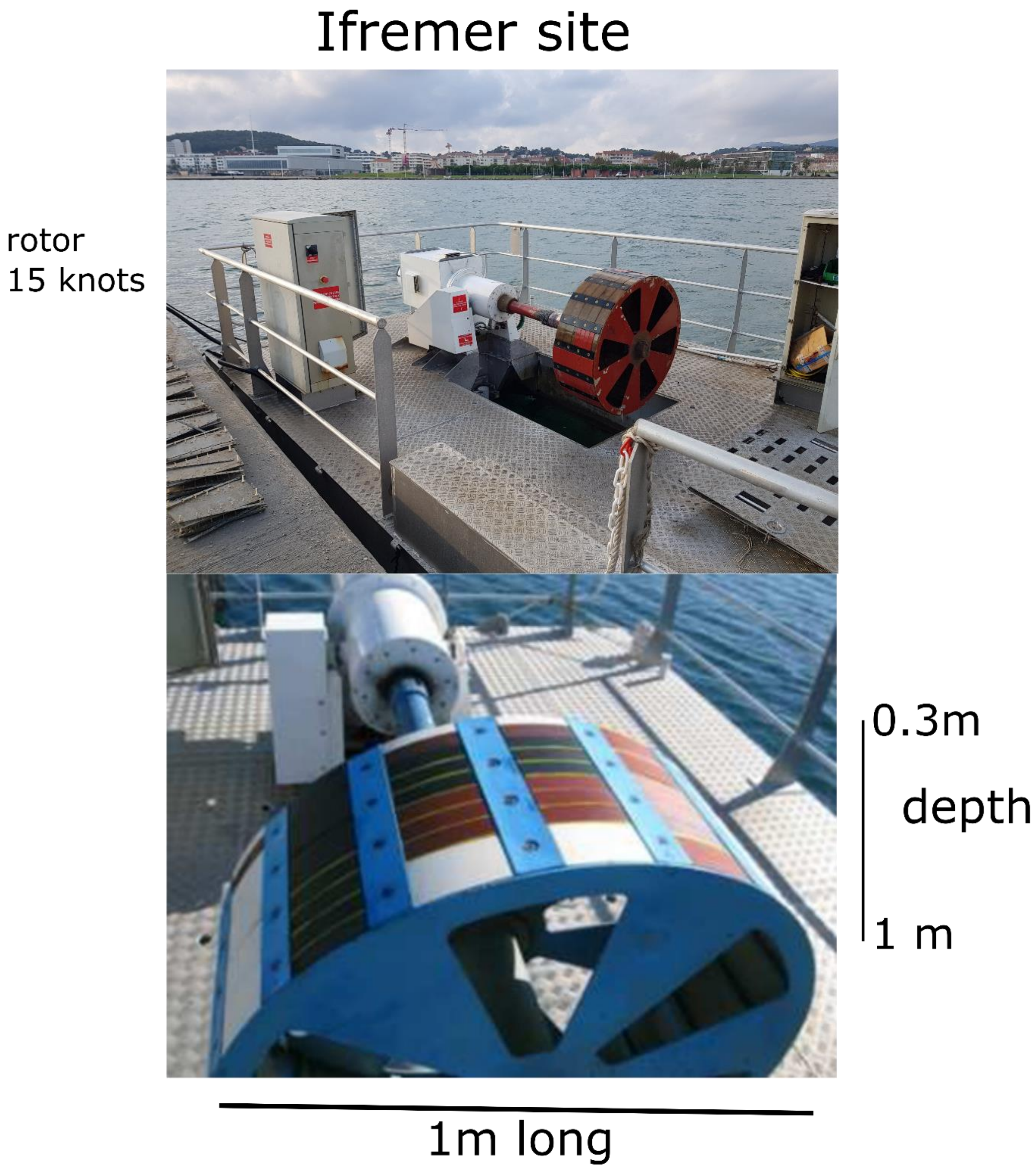

Figure S1. Photography of rotor and the dynamic immersion method. Upper picture displays the barge where the rotor is sustained. Rotor on horizontal position for inspection of plates. Lower picture shows in closer look the dimensions of the rotor and the disposition of the plates.

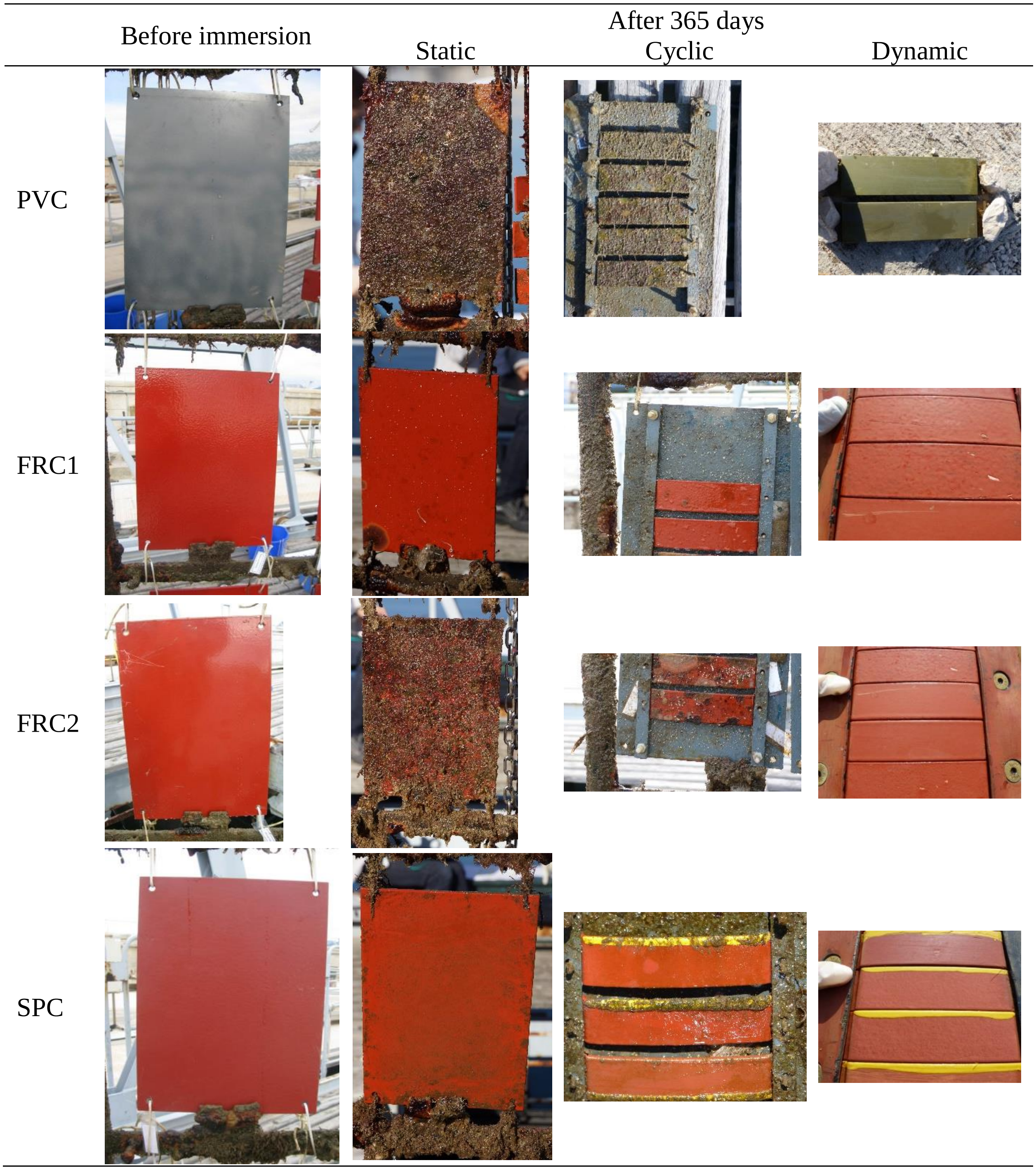

Figure S2. Photography of surfaces before and after (365 days) incubation.

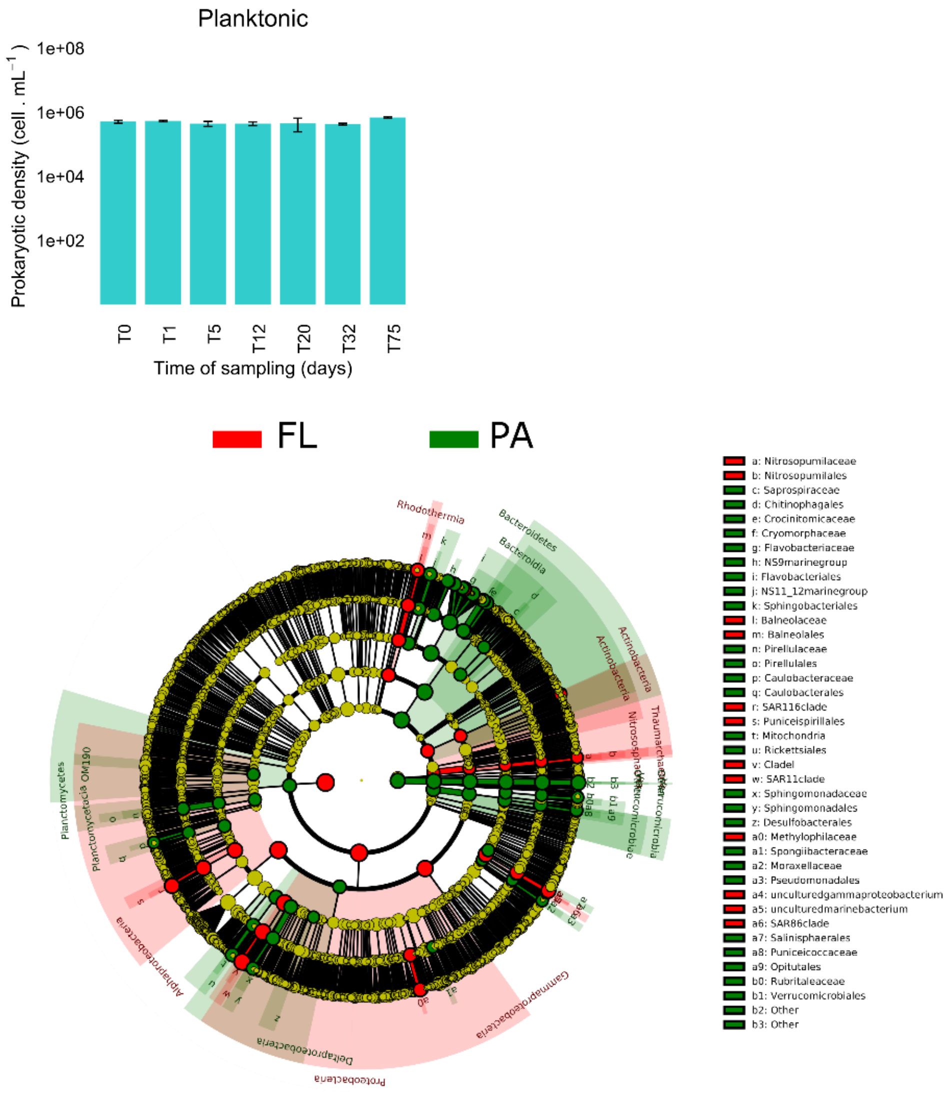

Figure S3. A) Evolution of planktonic cell density of the heterotrophic prokaryotes over time. Error bars represent standard errors. No difference was found between timepoints. B) LEfSe analysis cladogram identified discriminant taxa between FL (red) and PA (green) communities. Only taxa with LDA > 2.0 are displayed. FL – Free Living; PA – Particle Attached.

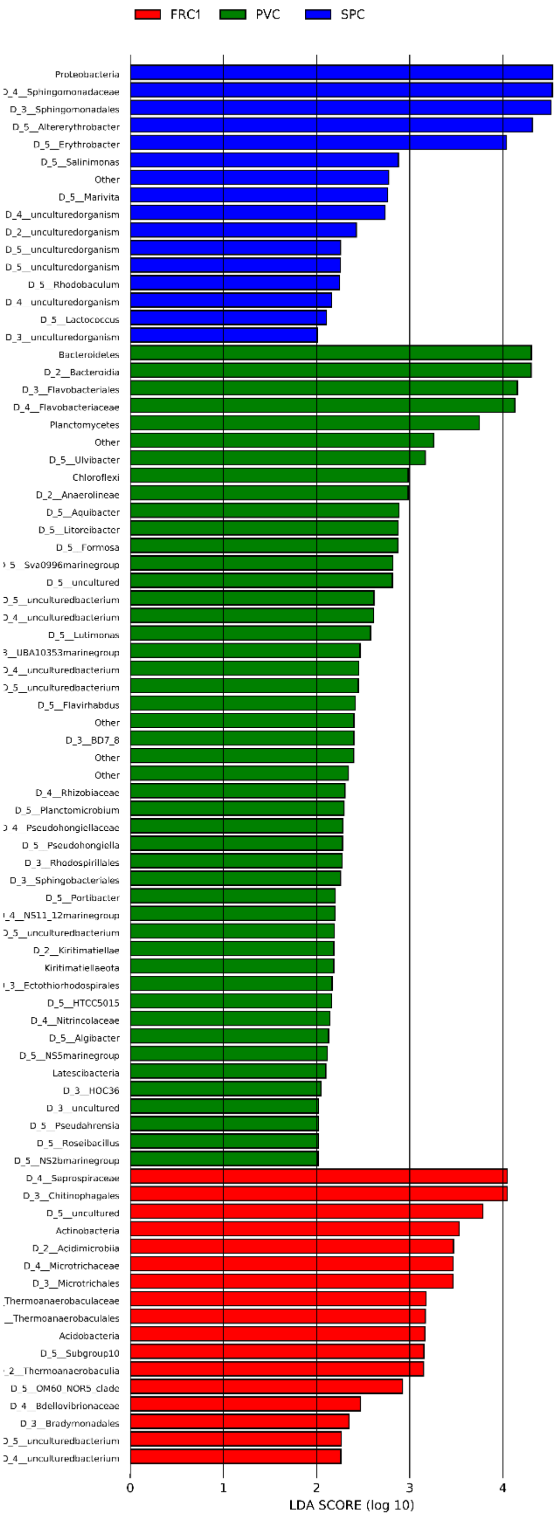

Figure S4. LDA Score of the discriminant taxa detected by LEfSe on the three types of surface incubated in dynamic mode. Different phylogenetic levels are represented in the y-axis, depending on the level of discrimination detected by the analysis.

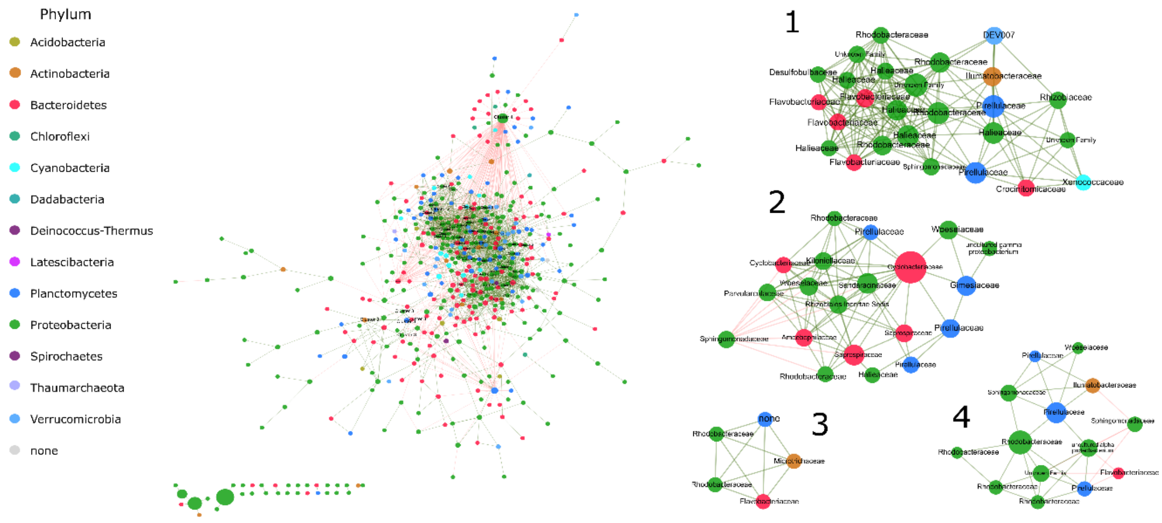

Figure S5. Network analysis on OTUs detected on PVC, FRC1, FRC2 and SPC immersed in four modes (dynamic, cyclic, static-Toulon and static-Banyuls) after one year (2455 OTUs) formed 507 nodes connected by 1988 edges (79% positive and 21% negative correlations). Positive (green) and negative (red) Spearman correlations ($P<0.05$; Benjamini-Hochberg multiple comparison correction). Node size is proportional to the betweenness centrality (non-directional network analysis), and nodes are colored by phylum. Network topology is shown in general, as well as specific clusters obtained with MCODE (1-4). Clusters 1, 3 and 4 represented OTU interactions mainly in static modes. OTUs in cluster 1 were detected 32x less in dynamic mode, and in greatest abundances in Static-Banyuls. Both clusters 3 and 4 were representative of PVC and FRC surfaces, mainly in static mode. Only the Cluster 2 presented similar relative abundances in all incubation modes.

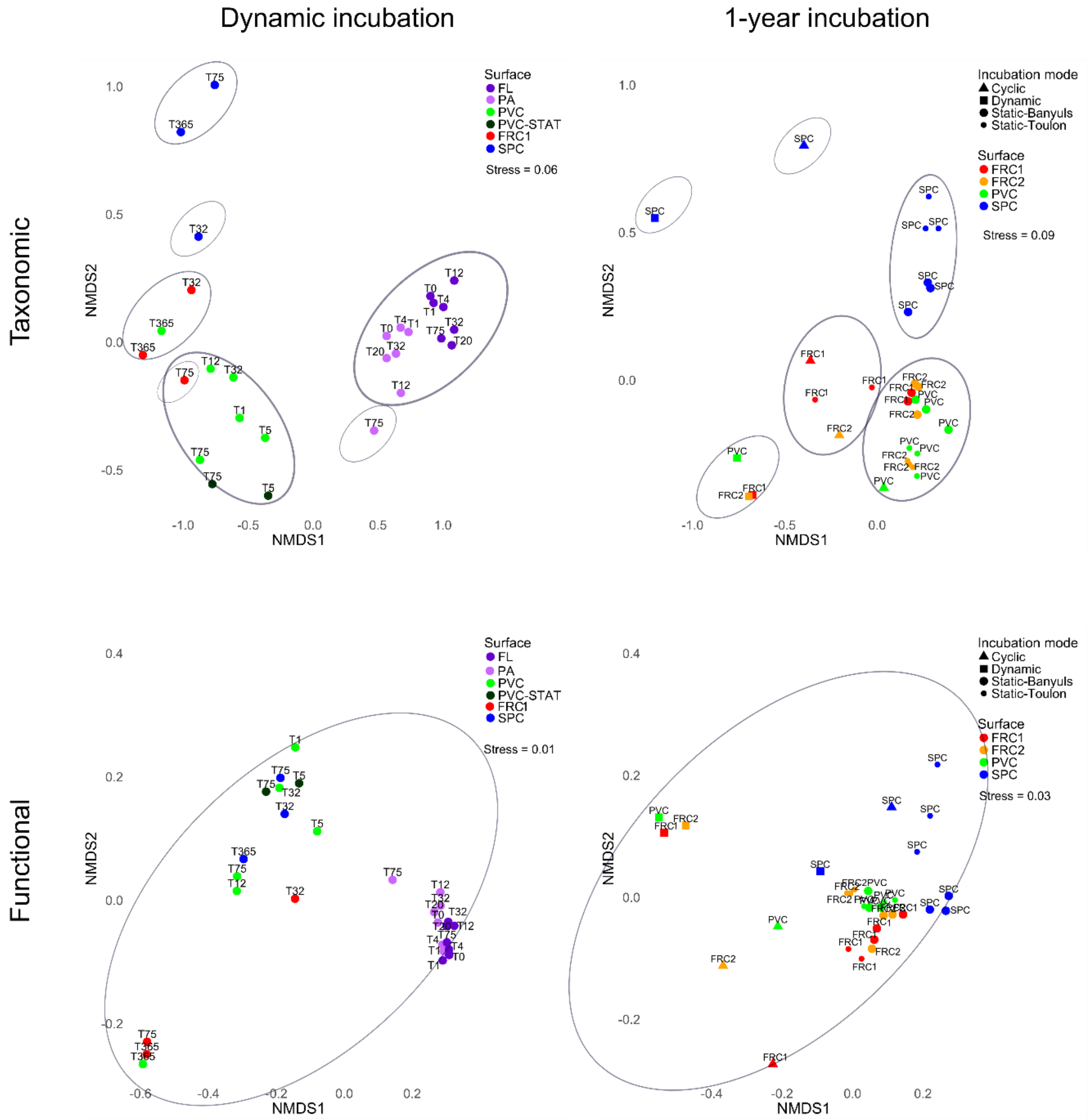

Figure S6. NMDS representation of Bray Curtis dissimilarity showing clusters (over 50% similarity) formed by samples according to surface or incubation mode. Upper figures were calculated from taxonomic OTU table(taxonomic) and lower figures from the table of predicted functions using the same OTU table as input (functional). Left figures represent analysis from three surfaces incubated in dynamic mode against the planktonic microbial community sampled at the same timepoints. Right figures represent comparisons made between the surfaces incubated under different modes after one year of incubation. Stress value under 0.1 denotes good ordination of microbial community samples in two dimensions. PERMANOVA p-value was equal to 0.001 in surface effect and/or incubation mode effect for taxonomic data. For functional data, surface effect was also significant (p 0.001), but after one year only incubation mode was under 0.01.

References

Bader, G. D., and Hogue, C. W. (2003). An automated method for finding molecular complexes in large protein interaction networks. *BMC Bioinformatics* 4, 2. doi:10.1186/1471-2105-4-2.

Caporaso, J. G., Kuczynski, J., Stombaugh, J., Bittinger, K., Bushman, F. D., Costello, E. K., et al. (2010). QIIME allows analysis of high-throughput community sequencing data. *Nature Methods* 7, 335–336. doi:10.1038/nmeth.f.303.

Cindrić, A.-M., Garnier, C., Oursel, B., Pižeta, I., and Omanović, D. (2015). Evidencing the natural and anthropogenic processes controlling trace metals dynamic in a highly stratified estuary: The Krka River estuary (Adriatic, Croatia). *Marine Pollution Bulletin* 94, 199–216. doi:10.1016/j.marpolbul.2015.02.029.

Coclet, C., Garnier, C., Delpy, F., Jamet, D., Durrieu, G., Le Poupon, C., et al. (2018). Trace metal contamination as a toxic and structuring factor impacting ultraphytoplankton communities in a multicontaminated Mediterranean coastal area. *Progress in Oceanography* 163, 196–213. doi:10.1016/j.pocean.2017.06.006.

Faust, K., and Raes, J. (2016). CoNet app: inference of biological association networks using Cytoscape. *F1000Res* 5. doi:10.12688/f1000research.9050.2.

Morley, N. H., Burton, J. D., Tankere, S. P. C., and Martin, J.-M. (1997). Distribution and behaviour of some dissolved trace metals in the western Mediterranean Sea. *Deep Sea Research Part II: Topical Studies in Oceanography* 44, 675–691. doi:10.1016/S0967-0645(96)00098-7.

Oursel, B., Garnier, C., Durrieu, G., Mounier, S., Omanović, D., and Lucas, Y. (2013). Dynamics and fates of trace metals chronically input in a Mediterranean coastal zone impacted by a large urban area. *Marine Pollution Bulletin* 69, 137–149. doi:10.1016/j.marpolbul.2013.01.023.

Schloss, P. D., Westcott, S. L., Ryabin, T., Hall, J. R., Hartmann, M., Hollister, E. B., et al. (2009). Introducing mothur: Open-Source, Platform-Independent, Community-Supported Software for Describing and Comparing Microbial Communities. *Applied and Environmental Microbiology* 75, 7537–7541. doi:10.1128/AEM.01541-09.

Schmieder, R., and Edwards, R. (2011). Quality control and preprocessing of metagenomic datasets. *Bioinformatics* 27, 863–864. doi:10.1093/bioinformatics/btr026.

Shannon, P., Markiel, A., Ozier, O., Baliga, N. S., Wang, J. T., Ramage, D., et al. (2003). Cytoscape: A Software Environment for Integrated Models of Biomolecular Interaction Networks. *Genome Res.* 13, 2498–2504. doi:10.1101/gr.1239303.

Smoot, M. E., Ono, K., Ruscheinski, J., Wang, P.-L., and Ideker, T. (2011). Cytoscape 2.8: new features for data integration and network visualization. *Bioinformatics* 27, 431–432. doi:10.1093/bioinformatics/btq675.

Tankere, S. P. C., and Statham, P. J. (1996). Distribution of dissolved Cd, Cu, Ni and Zn in the Adriatic Sea. *Marine Pollution Bulletin* 32, 623–630. doi:10.1016/0025-326X(96)00025-2.
